# The Easiest Children to Reach Are Most Likely to Be Infected with Ocular *Chlamydia trachomatis* in Trachoma Endemic Areas of Niger

**DOI:** 10.1371/journal.pntd.0001983

**Published:** 2013-01-10

**Authors:** Abdou Amza, Boubacar Kadri, Baido Nassirou, Sun N. Yu, Nicole E. Stoller, Satasuk J. Bhosai, Zhaoxia Zhou, Charles E. McCulloch, Sheila K. West, Robin L. Bailey, Jeremy D. Keenan, Thomas M. Lietman, Bruce D. Gaynor

**Affiliations:** 1 Programme National de Lutte Contre la Cecité, Niamey, Niger; 2 F.I. Proctor Foundation, University of California San Francisco, San Francisco, California, United States of America; 3 Department of Epidemiology & Biostatistics, University of California San Francisco, San Francisco, California, United States of America; 4 Dana Center for Preventive Ophthalmology, Wilmer Eye Institute, Johns Hopkins University, Baltimore, Maryland, United States of America; 5 Clinical Research Unit, Department of Infectious and Tropical Diseases, London School of Hygiene & Tropical Medicine, London, United Kingdom; 6 Department of Ophthalmology, University of California San Francisco, San Francisco, California, United States of America; 7 Institute for Global Health, University of California San Francisco, San Francisco, California, United States of America; Yale University, United States of America

## Abstract

**Background:**

Control programs for trachoma use mass antibiotic distributions to treat ocular *Chlamydia trachomatis* in an effort to eliminate this disease worldwide. To determine whether children infected with ocular *Chlamydia* are more likely to present later for examination than those who are uninfected, we compare the order of presentation for examination of children 0–5 years, and the presence of ocular *Chlamydia* by PCR in 4 villages in Niger where trachoma is endemic.

**Methods:**

We conducted a cluster-randomized, controlled trial where 48 randomly selected villages in Niger are divided into 4 study arms of different mass treatment strategies. In a substudy of the main trial, we randomly selected 1 village from each of the 4 study arms (4 total villages) and we evaluated the odds of ocular *Chlamydia* versus the rank order of presentation for examination and laboratory assessment before treatment was offered.

**Findings:**

We found the odds of harboring ocular *Chlamydia* dropped by more than 70% from the first child examined to the last child examined (OR 0.27, 95% CI 0.13–0.59, *P* = 0.001) in the 4 randomly selected villages. We found the odds of active trachoma dropped by 80% from the first child examined to the last child examined (OR 0.20, 95% CI 0.10–0.4, *P*<0.0001) in the 48 villages in the main trial.

**Interpretation:**

This study demonstrates that even if the WHO recommended 80% treatment coverage is not reached in certain settings, children 0–5 years with the greatest probability of ocular *Chlamydia* have higher odds of receiving attention because they are the first to present. These results suggest there may be diminishing returns when using scarce resources to track down the last few children in a mass treatment program.

**Trial Registration:**

ClinicalTrials.gov NCT00792922

## Author Summary

Trachoma is the most common cause of blindness from an infection in the world. The bacterium that causes trachoma is called *Chlamydia trachomatis* and it can be treated with the antibiotic azithromycin. Experts recommend trying to reach at least 80% of children for treatment in a community but it is unknown if this is necessary. We began a clinical trial in Niger in 48 villages in the summer of 2010 with mass drug administration (MDA) of azithromycin. We found that the odds of an eye infection were the highest in the first children to come for an examination. This means the extra time and money needed to reach all of the children in a village may provide diminishing returns because the easiest children to reach have the highest odds of infection. Perhaps it would be better to try to reach more villages for MDA instead of spending a lot of time and money trying to reach every single child in every single village.

## Introduction

Mass drug administration with oral azithromycin is known to be effective against trachoma, a blinding eye disease caused by ocular *Chlamydia trachomatis*. To achieve global elimination by 2020, the WHO recommends district-level mass treatment with antibiotics at 80% coverage for a minimum of 3 years, if the prevalence of clinical disease exceeds 10% prevalence in 1–9 year olds [1]. The 80% coverage recommendation is based on expert opinion rather than existing data; however, achieving target treatment coverage can be difficult in some settings [2], [3]. Children who are more difficult to locate for examination may be more likely to be infected with *C. trachomatis*, and using additional resources to reach these children may be even more important. In this study of 48 villages in Niger, we assess whether children aged 0–5 years who present late for examination and treatment have equal odds of active trachoma as those who present early. In a substudy of 4 villages in Niger, we assess whether children aged 0–5 years who present late for examination and treatment have equal odds of PCR-detected ocular *Chlamydia* as those who present early.

## Methods

### Study Setting and Design

The WHO recommends a single dose of oral azithromycin 20 mg/kg up to 1 gram for residents over 1 year of age; infants 1 year and younger are offered topical tetracycline [1], [4]. To determine whether children who present early for examination are more likely to be infected with ocular *Chlamydia* than children who present late for examination, we performed a substudy nested within a cluster-randomized clinical trial (PRET, Partnership for the Rapid Elimination of Trachoma) in the Matameye district of the Zinder region of Niger. The details of PRET have been previously described and are briefly summarized here [3], [5]. For the PRET trail, a total of 48 *grappes* (government health units, referred to as *villages* in this manuscript) were randomized to 4 arms with different mass azithromycin treatment strategies. The 48 villages were selected from among 6 health centers (Centre de Santé Intégrée or CSIs) and were eligible for inclusion if they had an estimated total population of between 250 to 600 persons, generally corresponding to 50 to 100 children in the eligible age range; distance >4 kilometers from the center of any semi-urban area because communities which are close to urban population centers are believed to have a lower prevalence of trachoma; and prevalence of active trachoma (TF using the WHO system) ≥10% in children aged 0–5 years.

**Figure 1:**
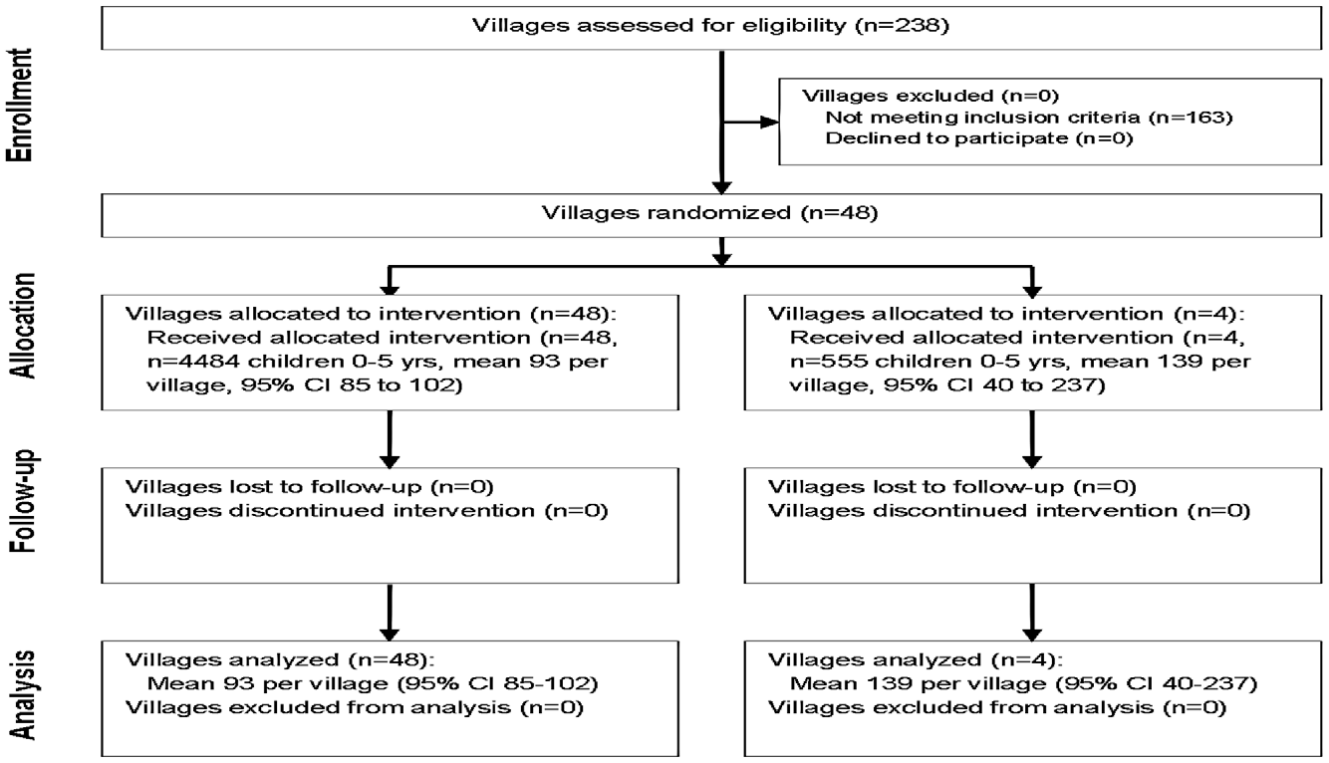
Consort flow diagram for cluster randomized trial.

Two separate assessments were performed: an analysis of active trachoma in 48 villages, and an analysis of both active trachoma and PCR-detected ocular *Chlamydia* in 4 substudy villages (Figure 1). If there were fewer than 100 children aged 0–5 years in one of the 48 villages, all children were included. If there were more than 100 children aged 0–5 years in one of the 48 villages, a random sample of 120 children was generated as the sentinel group for inclusion (note the target was ≤100 children but 120 was chosen to maximize the chance of obtaining 100 for the study). In the 4 substudy villages, all children aged 0–5 years were included regardless of village size. Randomization of communities and individuals was done using RANDOM and SORT functions in Excel (Version 2003) by BN. Only pretreatment results are presented here.

### Data Collection and Intervention

A pretreatment population census was completed in all villages in April 2010 by trained local personnel. Prior to examination, parents and guardians were notified by a village mobilizer to bring children 0–5 years of age to a central village location at a prespecified time for ocular examination and conjunctival swabbing for ocular *Chlamydia* PCR. Eligible children had a single opportunity for examination and field workers recorded the consecutive order in which the children presented. After obtaining consent from a parent or guardian, clinical grading of the right everted superior tarsal conjunctiva was performed using the WHO grading system (TF) by certified graders, with a 2.5× magnifying loupe and torch light or adequate sunlight [5], [6]. Following conjunctival examination, a Dacron swab was passed firmly 3 times over the right upper tarsal conjunctiva, rotating 120 degrees between each pass in all villages, as described previously [5]. Examiners changed gloves before examining each new participant. All of the samples were placed immediately at 4°C in the field and frozen at −20°C within 10 hours. Swabs were shipped at 4°C to University of California, San Francisco, CA, USA, where they were stored at −80°C until processing [7]. The Amplicor PCR assay (Roche Diagnostics, Branchburg, NJ, USA) was used to detect *C. trachomatis* DNA.

For the primary analysis, individual-level data for ocular *Chlamydia* were available in the 4 substudy villages. In the main study of 48 villages, swabs for ocular *Chlamydia* PCR were pooled after collection for community-level analysis to save time and cost as previously described [8], [9]. This pooling process for PCR precludes analysis of individual-level infection data; however, individual-level data for active trachoma were available in all 48 villages, and were included in a secondary analysis. Throughout the study, field workers were masked to ocular *Chlamydia* and laboratory workers were masked to field grade. The census, clinical exams, and swabbing for ocular *Chlamydia* PCR were all part of this research study and were performed outside of any existing mass distribution programs.

### Statistical Analysis

We normalized presentation rank by dividing presentation rank by the total number of children 0–5 years in each of the villages, to avoid larger villages from exerting more weight in the analysis. For the primary question, we estimated the odds of positive PCR given the normalized rank order of presentation for examination using mixed effects logistic regression with village and household as random effects. As a sensitivity analysis, we performed the same logistic regression with village as a fixed effect. For a secondary question, we did similar estimates with active trachoma as the outcome variable in all 48 villages using mixed effects logistic regression with village and household as random effects. All statistical analyses were performed with STATA 11 (College Station, TX).

### Human Participants and Consent Procedures

Ethical approval for this study was obtained from the Committee for Human Research of the University of California, San Francisco and le Comité Consultatif National d'Ethique du Ministère de la Santé Publique, Niger (Ethical Committee, Niger Ministry of Health).Verbal consent was approved by the IRB due to the high illiteracy rates in the study area and obtained from the community leaders before examination. Verbal consent was also obtained from the child's parent or guardian at the time of examination and was documented on the registration form for each study participant prior to examination in the field. The study was carried out in accordance with the Declaration of Helsinki.

## Results

All measurements were performed in June and July 2010. In the 4 substudy villages, there were a total of 555 children aged 0–5 years enrolled with a mean of 139 (range 58 to 196) per village (Table 1). The mean age of 0–5 year old children was 2.7 years (95% CI 2.6 to 2.9) per village and 51.1% (95% CI 47.2 to 54.8) were boys. The mean village prevalence of active trachoma was 23.9% (95% CI 8.5 to 39.2) and the mean village prevalence of ocular *Chlamydia* by PCR was 18.7% (95% CI 3.0 to 34.3) in these 4 substudy villages (Table 2). In the larger study of 48 villages, there were a total of 4484 children aged 0–5 years enrolled with a mean of 93.4 children per village (95% CI 85.2 to 101.6). The mean age of 0–5 year olds was 2.7 years (95% CI 2.7 to 2.8) in each village and 50.6% (95% CI 49.4 to 51.8) were boys. The mean prevalence of active trachoma was 24.8% (95% CI 20.8 to 28.8) per village.

**Table 1:** Village-level characteristics of 4 treatment villages in Niger.

	Village				
	A	B	C	D	Mean 4 villages (95% CI)
Total population	287	438	768	277	568 (178 to 957)
No. households	49	37	106	25	54 (3 to 111)
No. children 0–5 yrs	58	124	196	177	139 (40 to 237)
Mean age 0–5 yr olds	2.7	2.9	2.6	2.8	2.7 (2.5 to 3.0)
% households >30 min to water	34.7	5.4	0.0	0.0	10.0 (−0.2 to 0.4)
% households with latrine	0.0	0.0	5.7	57.3	15.7 (−0.3 to 0.6)
Fraction male	48.3	54.0	50.5	51.4	51.1 (47.2 to 54.8)

Mean (95% CI) unless otherwise noted.

**Table 2:** Odds ratios for active trachoma and ocular *Chlamydia* by normalized rank order.

Village	Active trachoma*	Ocular *Chlamydia* #	OR for ocular *Chlamydia* based on normalized rank order (95% CI)
A	19.0 (8.6 to 29.4)	8.6 (1.2 to 16.0)	0.03 (0.0005 to 1.97)
B	20.2 (13.0 to 27.3)	26.6 (18.1 to 34.5)	0.18 (0.04 to 0.75)
C	38.3 (31.4 to 45.1)	27.6 (21.2 to 33.9)	0.31 (0.10 to 0.99)
D	18.1 (12.4 to 23.8)	11.8 (7.1 to 16.7)	0.66 (0.13 to 3.37)
Mean 4 villages	23.9 (8.5 to 39.2)	18.7 (3.0 to 34.3)	0.27 (0.13 to 0.59)

* Trachomatous inflammation follicular (TF) according to WHO simplified trachoma grading system [6].

# Amplicor PCR (Roche Diagnostics, Branchburg, NJ, USA).

Community means (95% CI).

**Figure 2:**
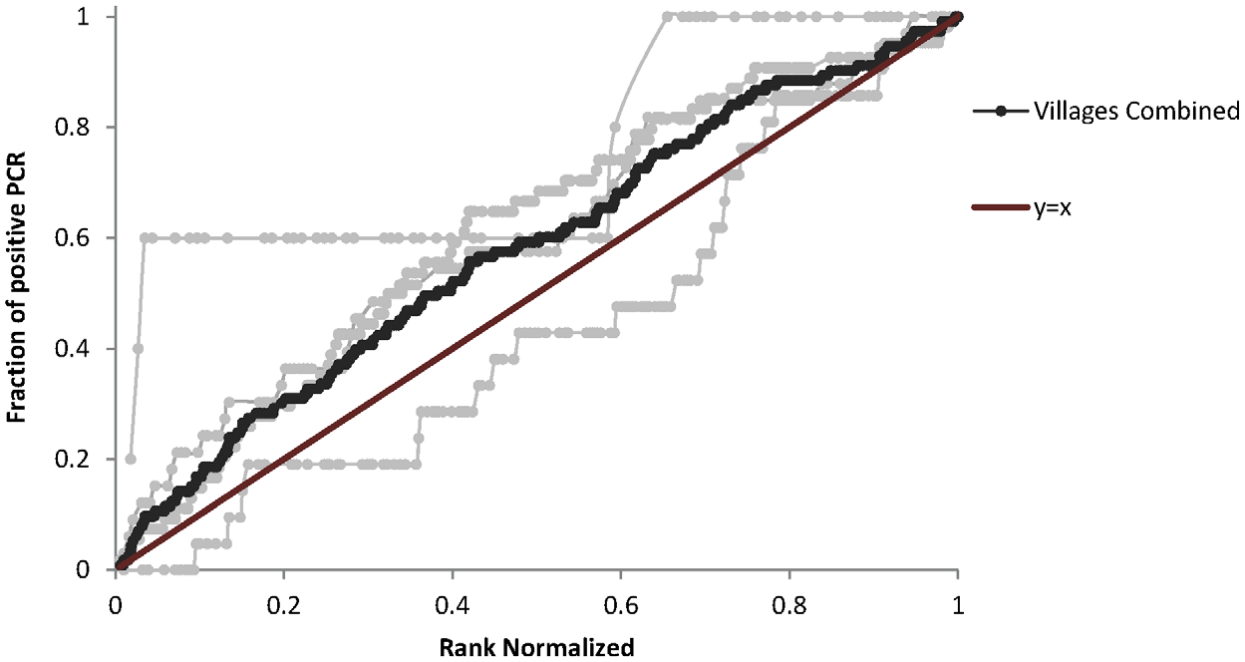
Rank of presentation (normalized) versus cumulative ocular *Chlamydia* PCR positivity aggregated in 4 villages in Niger. Infected individuals presenting earlier are displayed above y = x line.

**Figure 3:**
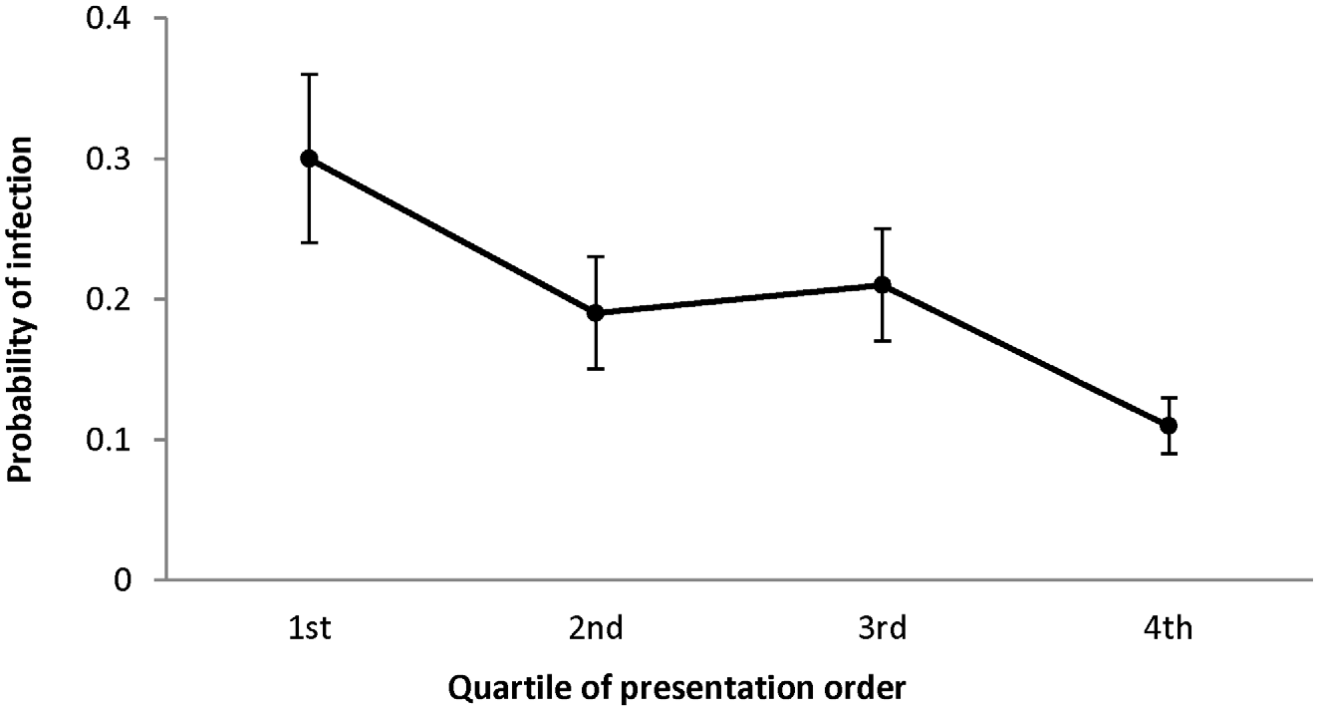
Probability of *C. trachomatis* infection by quartile of normalized rank order of presentation in 4 villages in Niger.

The odds of harboring ocular *Chlamydia* dropped in the 4 substudy villages by more than 70% from the first to the last child examined (OR 0.27, 95% CI 0.10 to 0.75, *P* = 0.01) in regression analysis (Figure 2). The results were similar with village as a fixed effect (OR 0.23, 95% CI 0.08 to 0.64, *P* = 0.005). When an individual presented in the next highest rank order quartile, the odds of ocular *Chlamydia* dropped by more than 25% (OR 0.73, 95% CI 0.60 to 0.88) using clustered logistic regression with village as a random effect (Figure 3). The odds of active trachoma were 80% less (OR 0.20, 95% CI 0.10 to 0.4, *P*<0.0001) for the last child compared to the first child examined. When we controlled for age and gender in the clustered logistic regression models, there was no substantial change in the results for the association of rank order and ocular *Chlamydia*. Finally, we found that an additional year in age (OR 0.54, 95% CI 0.21 to 1.35) and male gender (OR 1.64, 95% CI 0.92 to 2.93) were not predictive of rank order.

In the analysis of all 48 villages where clinical data were available for individuals, the odds of active trachoma dropped by 55% from the first to the last child examined (OR 0.45, 95% CI 0.33 to 0.61, *P*<0.0001) using clustered logistic regression with village and family as random effects. The results were similar with village as a fixed effect (95% CI 0.31–0.61), and there were no significant differences in the analysis when controlling for age or gender.

## Discussion

We found that children aged 0–5 years who present earlier for examination and testing prior to treatment for trachoma have higher odds of ocular *Chlamydia* than those who present later, in 4 villages in trachoma-endemic Niger. The odds of ocular *Chlamydia* dropped with each successive quartile of presentation. Researchers and program managers have been concerned that children in more disadvantaged families are more likely to harbor ocular *Chlamydia* and less likely to be brought earlier for examination. Here, we found the opposite—children with ocular *Chlamydia* are more likely to be brought earlier. Even if the WHO 80% treatment coverage goals are not reached in certain settings, the children who form the known reservoir of infection have higher odds of receiving attention because they are the first to present. There are several potential reasons to explain why early presenters have higher odds of harboring active trachoma and ocular *Chlamydia* than late presenters. Although active trachoma is believed to be a relatively mild or asymptomatic condition, parents and guardians may identify factors in their children such as intermittent ocular discharge or conjunctivitis which make them more likely to be brought for attention [10]. Active trachoma is spread within families and schools and can be exacerbated by overcrowding [11], [12]. It is reasonable to assume that children who are more social are more likely to be brought early for examination in a group setting where examinations are performed in Niger.

The extra effort required to locate individuals who are absent for examination may not be as important as had previously been thought. It may be practical to forgo pursuing difficult to reach children because the critical ‘core or herd’ group is present in the earlier quartiles, and coverage of infected persons is achieved prior to achieving coverage of the overall population.

Mathematical models and empirical studies suggest that disease elimination can be achieved even without treating all infected individuals in a community over time [9], [13], [14]. If a core group of infected individuals are repeatedly treated, then a degree of herd protection can be offered even to those who have not received treatment. Models assume that those untreated are no more likely to be infected than those treated. Here we find that those last to be examined are not *more* likely to be infected— they're *less* likely to be infected.

Although this study does not include data about expenses, it suggests an alteration of the cost-benefit equation in that the benefit of pursuing the final quartile of ‘hard-to-reach’ children has diminished value because it contains fewer infected children. Delivery of mass antibiotic treatment is expensive, especially in areas that are hard to reach [15], [16]. Even if oral azithromycin is donated, the extra effort required to locate absent individuals may be considerable. Our report supports results from an Ethiopian study that showed children examined on subsequent days after the initial monitoring day were less likely to have ocular *Chlamydia* than children seen on the initial day in an area hyperendemic for trachoma [17]. Antibiotic coverage is an important short-term predictor of ocular *Chlamydia* but may not be as important by six months after treatment, calling into question the necessity of achieving high coverage at all costs [18].

This study has some limitations. First, only 4 villages were included in the analysis of infection, and these villages may not be representative of the larger population in Niger or other trachoma-endemic countries. However, we found similar results in the 4 villages for rank order and infection as we did in the 48 villages for rank order and clinical disease. Second, only children aged 0–5 years were included, and there are children older than 5 years or adults in the communities who harbor infection. However, young children are known to be the most important reservoir of ocular *Chlamydia* in communities so exclusion of older children is unlikely to affect our estimates [9]. Third, this study was performed in a research setting which may not be generalizable to trachoma treatment programs that do not have the same resources. Finally, this was a study of the association of order of presentation with active trachoma and ocular *Chlamydia* PCR, not antibiotic treatment coverage. It is not unreasonable to assume that children who present earlier for examination are more likely to present earlier for treatment, but this was not measured in this study.

In summary, we found that children 0–5 years who have ocular *Chlamydia* have higher odds of presenting early for ocular examination than children who do not have ocular *Chlamydia* in Niger where trachoma is endemic. The added effort required to reach the last infected individuals in a community may provide diminishing returns. We suggest thoughtful use of limited health-care resources in trachoma programs, perhaps to reach more villages, rather than excessive efforts to attain high coverage in individual villages. Efficient use of limited resources is critical for the WHO to achieve their trachoma elimination goals by the year 2020.

## Supporting Information

Checklist S1 **CONSORT checklist.**
(DOCX)

Protocol S1 **Trial protocol.**
(PDF)
